# Advanced Facial Rejuvenation: Synergistic Effects of Lower Blepharoplasty and Ultrasound Guided Mid-Face Lift Using Polydioxanone (PDO) Threads

**DOI:** 10.1007/s00266-024-03975-6

**Published:** 2024-03-22

**Authors:** Jun Ho Park, Ji Won Jeong, Ji-Ung Park

**Affiliations:** Department of Plastic and Reconstructive Surgery, Seoul National University College of Medicine, SMG-SNU Boramae Medical Center, 20 Boramae-ro 5-gil, Dongjak-gu, 07061 Republic of Korea

**Keywords:** Lower blepharoplasty, Mid-face lift, PDO thread, Ultrasound

## Abstract

**Background:**

Traditional facial aging surgeries have risks and extended recovery times, leading to a demand for minimally invasive alternatives. PDO (polydioxanone) threads, which are absorbable sutures that stimulate collagen production and tissue contraction, offer improved aesthetic outcomes. This paper evaluates the combined use of PDO thread mid-cheek lift and lower blepharoplasty for facial rejuvenation.

**Methods:**

This retrospective study compared outcomes in patients undergoing lower blepharoplasty combined with a mid-face lift using PDO threads versus those undergoing only lower blepharoplasty. Focused on individuals with baggy lower eyelids and pronounced nasolabial folds, outcome measures included the Modified Fitzpatrick wrinkle scale, Allergan® midface volume deficit scale, Width of inter zygomatic distance, Patient and Observer Scar Assessment Scale, and patient satisfaction questionnaires, assessed at baseline, 3 months, and 1 year postoperatively.

**Results:**

The combined procedure demonstrated superior aesthetic outcomes and higher patient satisfaction compared to lower blepharoplasty alone. Improvements were more significant in wrinkle reduction, midface volume, and inter-zygomatic distance in the combined procedure group. Although the combined procedure had a longer mean operation time, scar assessment scores were similar between both groups, with no complications reported.

**Conclusion:**

The combination of lower blepharoplasty and mid-face lift using PDO threads is a comprehensive and effective approach for facial rejuvenation. It significantly enhances wrinkle reduction, mid-face lifting, and patient satisfaction. Ultrasound-guided thread lifting, a method of assessing and performing mid-face lifting, proves to be safe and efficient. This approach holds promise as a future option in cosmetic anti-aging surgery, presenting a minimally invasive alternative with natural-looking results and reduced downtime.

**Level of Evidence II:**

This journal requires that authors assign a level of evidence to each article. For a full description of these Evidence-Based Medicine ratings, please refer to the Table of Contents or the online Instructions to Authors https://link.springer.com/journal/00266.

## Introduction

Facial aging is a complex process involving changes in skin texture, volume loss, and gravitational descent of facial tissues [1]. Signs of aging, especially lower eyelid and mid-cheek laxity and descent, contribute to an "aged" and "tired" appearance, leading many individuals to seek corrective interventions [2]. Commonly employed surgical approaches, such as blepharoplasty and mid-cheek lift, tackle distinct aspects of the aging process [3]. However, these traditional procedures carry risks and require substantial recovery time, underscoring the need for minimally invasive, efficient, and effective alternatives [4].

In recent times, there has been a significant uptick in the demand for minimally invasive aesthetic procedures [5]. The mid-cheek region, located between the lower eyelid and the upper lip, is often among the first facial areas to exhibit aging signs, such as sagging cheeks, pronounced nasolabial folds, and baggy lower eyelids [6]. Traditional mid-cheek lifts involve surgical procedures posing certain risks, recovery time, and potential scarring to patients [7]. This has led to a growing interest in developing less invasive but effective techniques among practitioners and patients.

Absorbable sutures, such as Polydioxanone (PDO) threads, have gained considerable traction in the aesthetic field recently. This innovative, minimally invasive technique holds promise for tissue suspension and rejuvenation. PDO threads stimulate a selective inflammatory response that encourages collagen production and tissue contraction, resulting in enhanced aesthetic outcomes. [8, 9] The benefits of this method include less downtime, reduced risk of complications, and the capability to achieve natural-looking results.

Blepharoplasty, a surgical procedure to rectify age-related eyelid deformation and aesthetically enhance the periorbital area of the face, is one of the most commonly performed cosmetic plastic surgery in the United States. [10] Lower eyelid blepharoplasty, in particular, addresses both functional and aesthetic issues in the area from the lower eyelid to the upper part of the cheek by excising skin, repositioning adipose tissue, and reinforcing the periorbital muscle, including the canthal tendon [11]. However, while patient satisfaction can be enhanced through various surgical techniques, their effectiveness on the mid-face can be limited, and side effects such as ectropion and lagophthalmos may occur due to excessive skin resection. Other methods, like laser treatment and surgical lifts for mid-face rejuvenation, also have their limitations due to minor effects or scarring from skin incisions. [12]

As an alternative, absorbable thread lifting with PDO threads, a common and safe material in minimally invasive aesthetic procedures, has emerged. This innovative procedure promises to address these challenges, offering a non-surgical solution for facial rejuvenation. [13] The technique uses bio-absorbable threads to lift sagging facial tissues and stimulate collagen synthesis for enduring effects. Although thread lifting has been extensively used for overall facial rejuvenation, its application specifically for a mid-cheek lift is yet to be thoroughly investigated. This paper introduces a novel combined approach of lower blepharoplasty and mid-cheek lift using PDO threads, offering a comprehensive solution for facial rejuvenation. The aim of this study is to describe the procedure in detail, evaluate its efficacy and safety, and assess patient satisfaction. This research adds to the burgeoning body of literature on minimally invasive techniques for facial rejuvenation, especially analyzing the effect of performing mid-face lift surgery using PDO thread in conjunction with lower eyelid blepharoplasty.

## Materials and Methods

Our institutional review board (IRB No. 20-2023-24) approved this retrospective review of lower blepharoplasty combined with mid-face lift using PDO thread. All patients included in this analysis provided written informed consent after 1 postoperative month at the outpatient clinic. The study was conducted in accordance with the Declaration of Helsinki and its later amendments. Informed consent was obtained from patients for all surgical procedures and wound management, and for the possible use of anonymized photographs. Eligible patients were those who underwent lower blepharoplasty for baggy lower eyelid with pronounced nasolabial fold from March 2019 through December 2022. The patients were thoroughly examined before the surgical procedure. Patients with poor general health, eye disorder such as glaucoma, thyroid disorder, severe dermatitis, and unrealistic expectations were excluded in this study. This was a single-center study, and a single attending plastic surgeon (J.H. Park) with 12 years of experience performed all procedures, which were carried out using standard methods.

### Technique

**Fig. 1:**
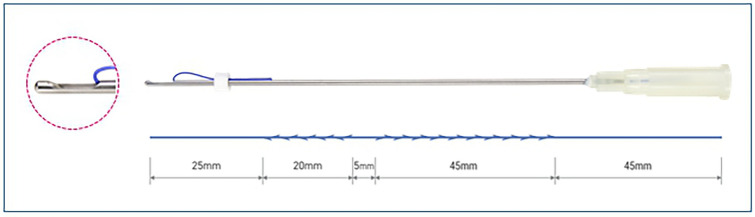
Single, cogged polydioxanone (PDO) thread with an attached 19-G catheter. The total length of the thread was 14cm. The 3-dimensional cog design had multiple barbs from bottom to top

**Fig. 2:**
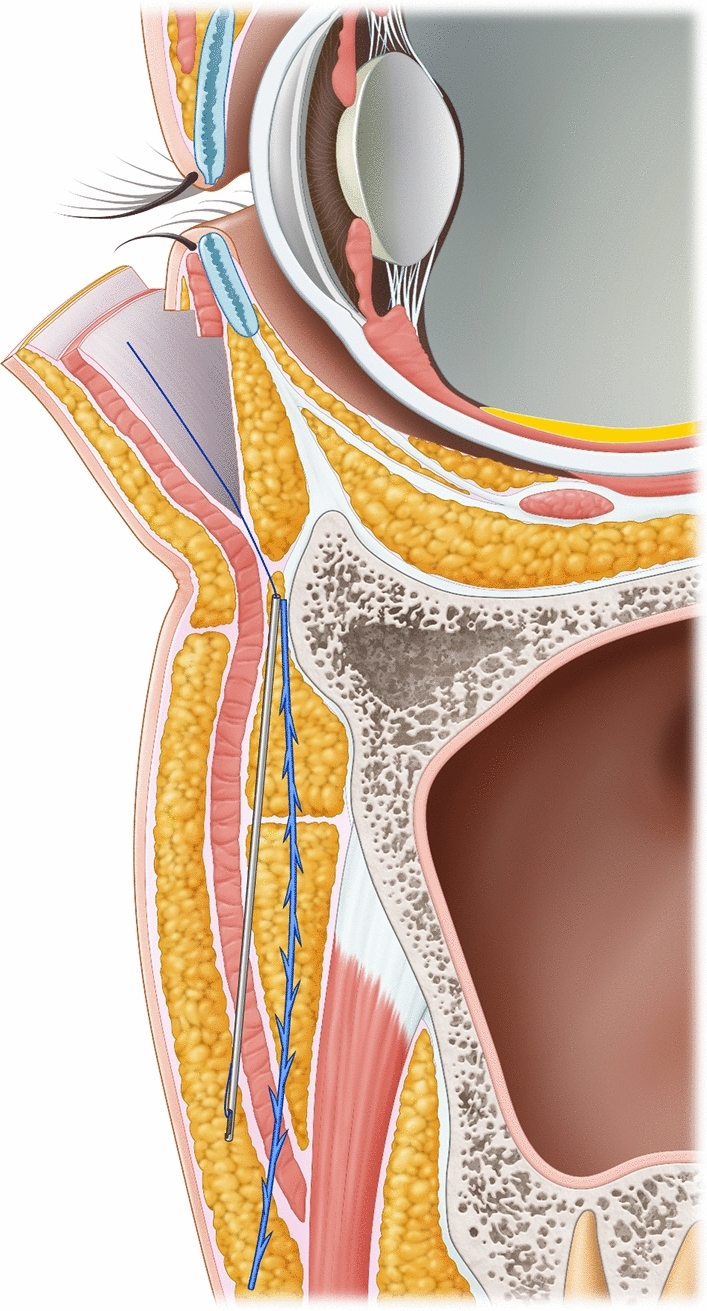
Schematic illustration of thread insertion. The cannula starts subperiosteal level from arcus marginalis at the entry point and advances into a more superficial direction, passing the deep fat pad and SMAS layer

**Fig. 3:**
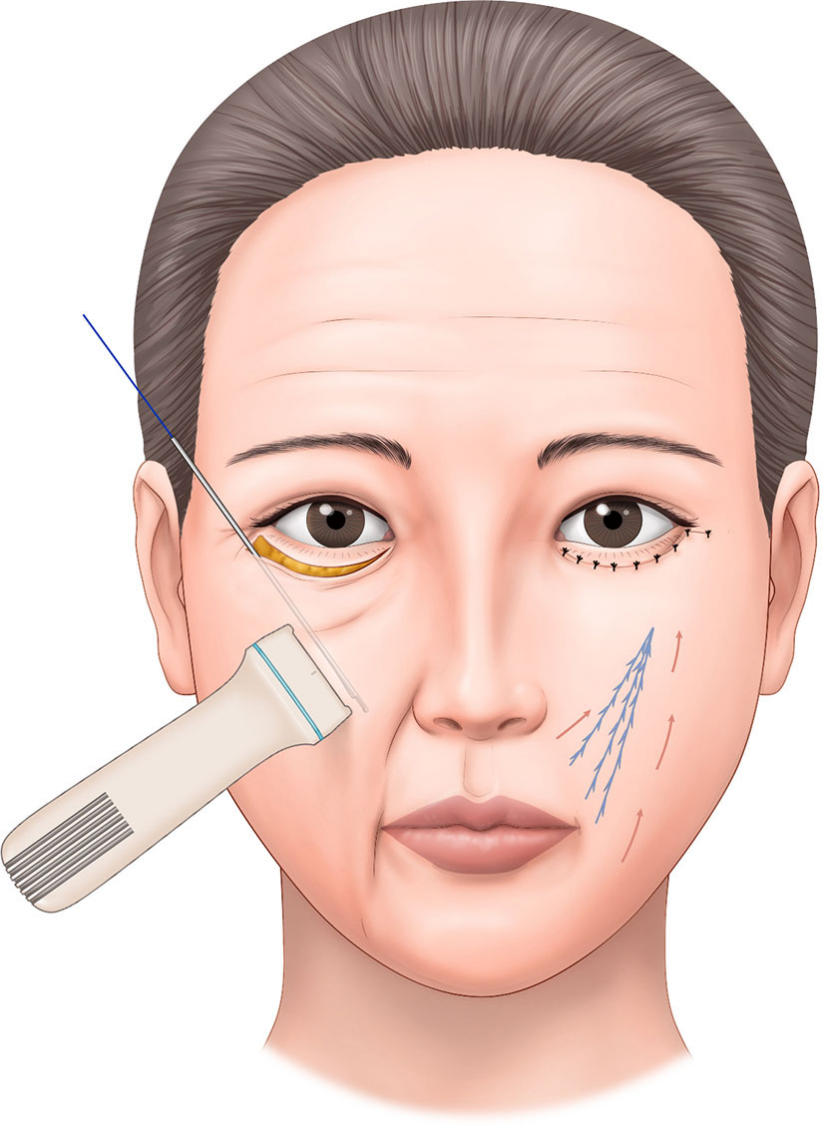
The thread-containing cannula is inserted after passing through the arcus marginalis. The insertion direction is guided towards the superficial layer, traversing the deep fat pad and superficial muscular aponeurotic system (SMAS) layer, and finally stopping superficially under the dermis, medially to the nasolabial fold, while utilizing portable handheld ultrasonography for guidance

**Fig. 4:**
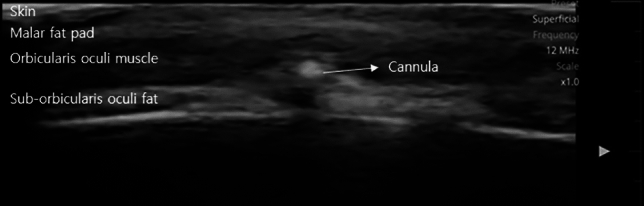
Intraoperative Ultrasonography finding demonstrate the trajectory of the cannula during its passage

The surgical procedure is performed under local anesthesia. A subciliary incision line is made on the lower eyelid. The skin is incised after injection of lidocaine mixed with epinephrine at a ratio of 1:100,000. Stepped skin-muscle flap dissection is carried out to reach the inferior orbital rim in all patients. Following identification and incision the orbital septum, redundant orbital fat is removed from the medial, central, and temporal compartments. The PDO cog thread (total length, 14cm) is pre-cannulated using a Medicut catheter type 19-G cannula (Mint PETIT; HansBiomed, Seoul, South Korea) (Fig. 1). Secured insertion through the arcus marginalis allows the catheter to be advanced into the anterior cheek, reaching the inferior level and passing through the nasolabial fold (Figs. 2, 3). As the cannula advances into the inferior level, the insertion direction is directed towards the superficial layer, passing through the deep fat pad and superficial muscular aponeurotic system (SMAS) layer, and stopping superficially under the dermis medial to the nasolabial fold under portable handheld ultrasonography (SONON 300L, Healcerion, Seoul, Korea) (Fig. 4). Once the cannula containing the thread is fully inserted, the cannula is removed, and the thread is kept in place. In all patients, three threads each side of cheek, in different directions from the superior to the inferior portion. After the removal of the cannula, the thread is pulled out of the skin using moderate force. At this point, the operator ensures that the threads are anchored to the arcus marginalis and orbital retaining ligament of the inferior orbit, as the anchoring resists the outward pulling force. After pulling the threads adequately parallel to the zygomaticus minor muscle, they are cut with sharp scissors. Subsequently, gentle manual pressure is applied over the cheek to release the dimpled area around the nasolabial fold.

After completion of the above mid-face lift procedure using PDO, lateral canthopexy is performed on each side in all patients using No. 4-0 Ethibond suture. The incised orbicularis oculi muscle is repaired with a single No. 5-0 PDS suture just below the lateral canthus, and skin closure is performed using No. 6-0 Nylon suture.

Upon completion of all the procedures, mild compressive foam dressing is applied to minimize swelling and bruising. The patients are instructed to keep their head elevated 30º during sleep for the initial day following the procedure. Antibiotic ointment is prescribed to be applied to the incision lines to prevent infection. Sutures are removed after a week during the postoperative follow-up.

### Outcome Measures

Each patient underwent preoperative and postoperative photography. Surgical outcome was assessed using the Modified Fitzpatrick wrinkle scale (MFWS), Allergan® midface volume deficit scale (AMVDS), Width of inter zygomatic distance of the midface to medial canthus (WIZDOM-MC), Patient and observer scar assessment scale (POSAS), and patient satisfaction questionnaire [14–17]. Outcome assessments were conducted preoperatively, three months post-operation, and one year after operation at the outpatient clinic. The MFWS was used to evaluate the characteristics and severity of nasolabial fold, with a grading system classified as follows: Class 0 (no visible wrinkle), Class 0.5 (very shallow yet visible wrinkle), Class 1 (visible wrinkle and slight indentation), Class 1.5 (visible wrinkle and clear indentation, less than 1mm wrinkle depth), Class 2 (moderate wrinkle, 1 to 2 mm wrinkle depth), Class 2.5 (prominent and visible wrinkle, 2 to 3mm wrinkle depth), and Class 3 (deep wrinkle, more than 3mm wrinkle depth). The AMVDS was assessed to evaluate changes in the midfacial region, with the scale classified as follows: Grade 0 (fullness in the zygomatic-malar region), Grade 1 (flattening in the zygomatic-malar region), Grade 2 (mild concavity in the zygomatic-malar region, mild tear troughs), Grade 3 (moderate concavity in the zygomatic-malar region, moderate tear troughs), Grade 4 (significant concavity in the zygomatic-malar region, moderate nasolabial folds), and Grade 5 (severe concavity in the zygomatic-malar region, significant nasolabial folds). The WIZDOM-MC was assessed by measuring the distance between the inter-zygomatic line and the medial canthus. The POSAS was assessed using 7 observer components and 7 patient components, with each component graded on a scale from 1 (good) to 10 (bad). To ensure accuracy, three blind observers specializing in plastic surgery evaluated each patient, and the mean scores were recorded. Additionally, to account for possible facial asymmetry, the severity of wrinkles on each side of the face was graded separately, and the mean scores were recorded. The scores at three months and one year after operation were calculated and recorded. Finally, for the subjective evaluation of surgical outcomes, patients were asked to complete a questionnaire regarding their level of satisfaction, using a 5-point grading system ranging from 1 (very poor) to 5 (very satisfied), both 3 months and 1 year postoperatively.

Statistical analyses were conducted by comparing the assessment scores at each visit using paired t-test methods, and descriptive statistics were reported as mean scale value and standard deviation (SD). The normality test was performed using the Kolmogorov-Smirnov test, and the analyses were conducted using SPSS Statistics for Windows, version 26.0 (IBM Corp., Armonk, NY, USA). Statistical significance was defined as p-values less than 0.05.

## Results

The surgical outcomes of lower blepharoplasty alone and lower blepharoplasty combined with mid-face lift were compared. A mean age was 64 years (range: 43–77 years) and the mean follow-up duration was 12.7 months. The total number of patients was 94 (48 for lower blepharoplasty alone and 46 for lower blepharoplasty with mid-face lift). Table 1 summarizes all other characteristics.

**Table 1:** Demographic findings of the patients

Criteria	Lower blepharoplasty only	Lower blepharoplasty with mid-face lift	*P*-value
Total number of patients	48	46	
Mean age (years)	63.4 ± 14.1	65.6±13.4	0.45
Mean follow-up period (months)	12.5 ± 0.2	12.8±0.4	0.37
*Gender*			
Male	20	22	0.64
Female	28	24	0.82
*Past surgical history*			
Lower blepharoplasty	3	2	0.62
Facial fat graft	0	0	0.84
Conventional face lift	1	1	0.71

The mean operation time was significantly longer for the combined procedure (60.2 ± 12.8 min) compared to the lower blepharoplasty alone (45.8 ± 11.6 min) with a *p*-value less than 0.01, suggesting a statistically significant difference (Table 2).

**Table 2:** Surgical outcomes of lower blepharoplasty only and Lower blepharoplasty with mid-face lift groups

	Lower blepharoplasty only	Lower blepharoplasty with mid-face lift	P-value
Total number of patients	48	46	
Mean operation time (min)	45.8 ± 11.6	60.2 ± 12.8	<0.01
Mean change of MFWS			
Initial	2.5 ± 0.3	2.4 ± 0.5	0.75
3 months	− 1.3 ± 0.4	− 1.8 ± 0.2	<0.01
1 year	− 0.9 ± 0.2	− 1.4 ± 0.2	<0.01
*Mean change of AMVDS*			
Initial	3.2 ± 0.5	3.4 ± 0.6	0.62
3 months	− 1.6 ± 0.3	− 2.2 ± 0.4	<0.01
1 year	− 1.0 ± 0.1	− 1.7 ± 0.2	<0.01
*Mean change of WIZDOM-MC (mm)*			
Initial	12.6 ± 1.4	13.1 ± 1.1	0.86
3 months	− 1.8 ± 0.6	− 3.4 ± 0.4	<0.01
1 year	− 1.4±0.1	− 2.7 ± 0.7	<0.01
*Mean POSAS*			
3 months	2.1 ± 0.4	2.2 ± 0.3	0.68
1 year	1.3 ± 0.3	1.2 ± 0.4	0.41
*Overall patient satisfaction*			
3 months	4.1 ± 0.6	4.6 ± 0.3	<0.01
1 year	3.4 ± 0.7	4.2 ± 0.5	<0.01

MFWS: Modified Fitzpatrick wrinkle scale

AMVDS: Allergan^®^ midface volume deficit scale

WIZDOM - MC: Width of inter zygomatic distance of the midface – medial canthus

POSAS: Patient and observer scar assessment scale

In terms of wrinkle scale changes as measured by the MFWS, the group that underwent lower blepharoplasty with mid-face lift showed greater improvements at both three months (− 1.8 ± 0.2 vs. − 1.3 ± 0.4) and one year (− 1.4 ± 0.2 vs. − 0.9 ± 0.2) post-operation compared to the group that underwent lower blepharoplasty alone. These differences were statistically significant with *p*-values less than 0.01.

Similar results were observed in the AMVDS and WIZDOM-MC, with the combined procedure group showing significantly greater improvements at three months and one year after operation. These differences were also statistically significant with *p*-values less than 0.01. The POSAS scores were similar for both groups at three months (2.1±0.4 vs. 2.2 ± 0.3) and one year (1.3 ± 0.3 vs. 1.2 ± 0.4) post-operation. The differences were not statistically significant with p-values of 0.681 and 0.412 respectively. Patient satisfaction was significantly higher in the combined procedure group at both three months (4.6 ± 0.3 vs. 4.1 ± 0.6) and one year (4.2 ± 0.5 vs. 3.4 ± 0.7) post-operation, with *p*-values less than 0.01. There were no complications, such as infection, hematoma, wound dehiscence, or skin irregularity reported in either group.

The results suggest that while both procedures were effective in improving aesthetic outcomes and patient satisfaction, the combined lower blepharoplasty with mid-face lift procedure demonstrated superior results despite a longer operation time. However, scar assessment did not significantly differ between the two groups.

Case #1

**Fig. 5:**
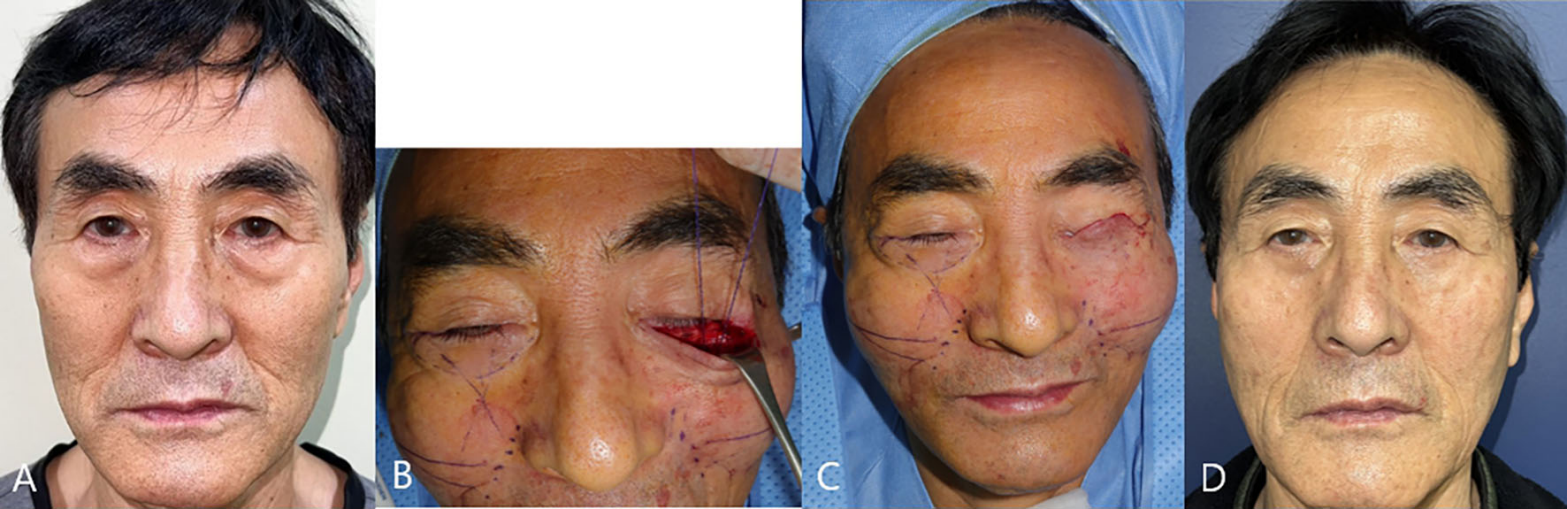
The first case of patient with lower blepharoplasty and mid-face lift using PDO threads. Photographic findings of 64-year-old male patient with baggy lower eyelid and aging process in mid-cheek region. **A** After redundant fat removal, three PDO threads were inserted. **B** Immediate postoperative photographic finding after mid-face lift. **C** Postoperative photographic finding; 1 year. **D**

A 64-year-old man visited clinic for a baggy lower eyelid and aging process in mid-cheek region. He had no history of facial aesthetic surgical procedure. The patient complained of soft tissue sagging in the anterior cheek and deeper-appearing nasolabial fold. Lower blepharoplasty including resection of redundant fat component and mid-face lift using three PDO threads were performed (Fig. 5). At one year after operation, mean changes of MFWS and AMVDS were 1.5 and 1.7. WIZDOM-MC decreased 2.6mm compared to preoperative finding and mean POSAS was 0.8 at 1 year after operation. Patient was very satisfied with the outcome of lower blepharoplasty combined with mid-face lift procedure.

Case #2

**Fig. 6:**
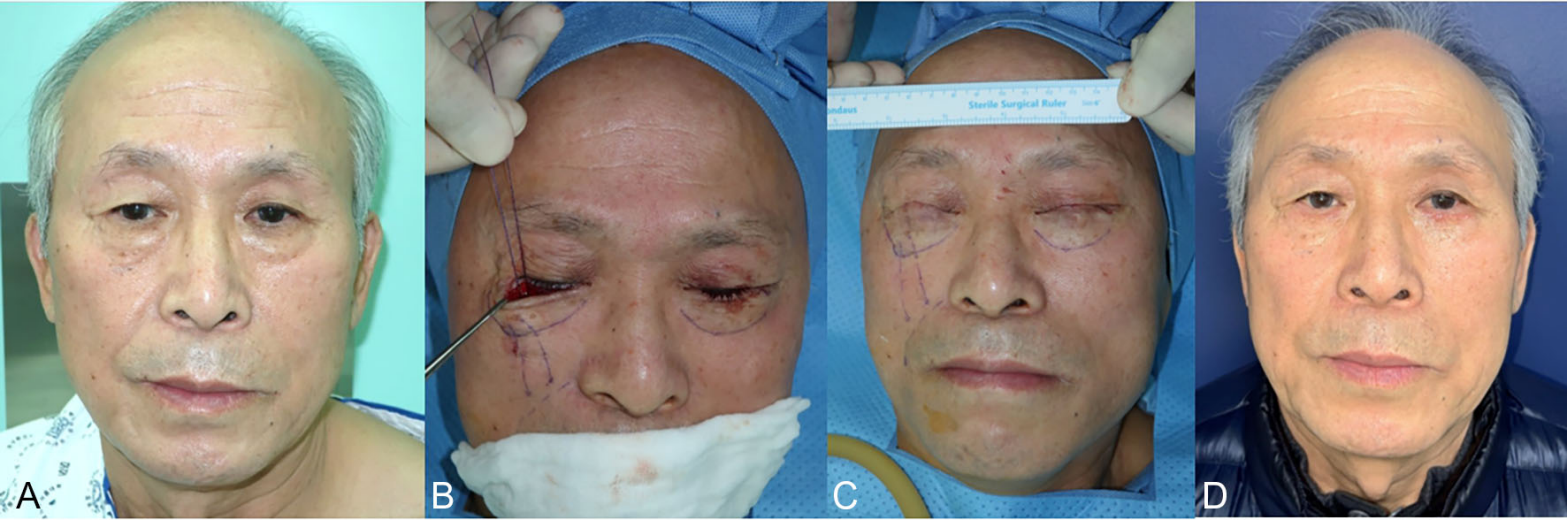
The second case of patient with lower blepharoplasty and mid-face lift using PDO threads. Photographic findings of 66-year-old male patient with baggy lower eyelid and aging process in mid-cheek region **A** After redundant fat removal, three PDO threads were inserted. **B** Immediate postoperative photographic finding after mid-face lift. **C** Postoperative photographic finding; 6 months. **D**

A 66-year-old man visited clinic for a baggy lower eyelid and aging process in mid-cheek region. He had no history of facial aesthetic surgical procedure. The patient complained of soft tissue sagging in the anterior cheek and deeper-appearing nasolabial fold. Lower blepharoplasty including resection of redundant fat component and mid-face lift using three PDO threads were performed (Fig. 6). At one year after operation, mean changes of MFWS and AMVDS were 1.2 and 1.6. WIZDOM-MC decreased 2.1mm compared to preoperative finding and mean POSAS was 1.1 at 1 year after operation. Patient was very satisfied with the outcome of lower blepharoplasty combined with mid-face lift procedure.

Case #3

**Fig. 7:**
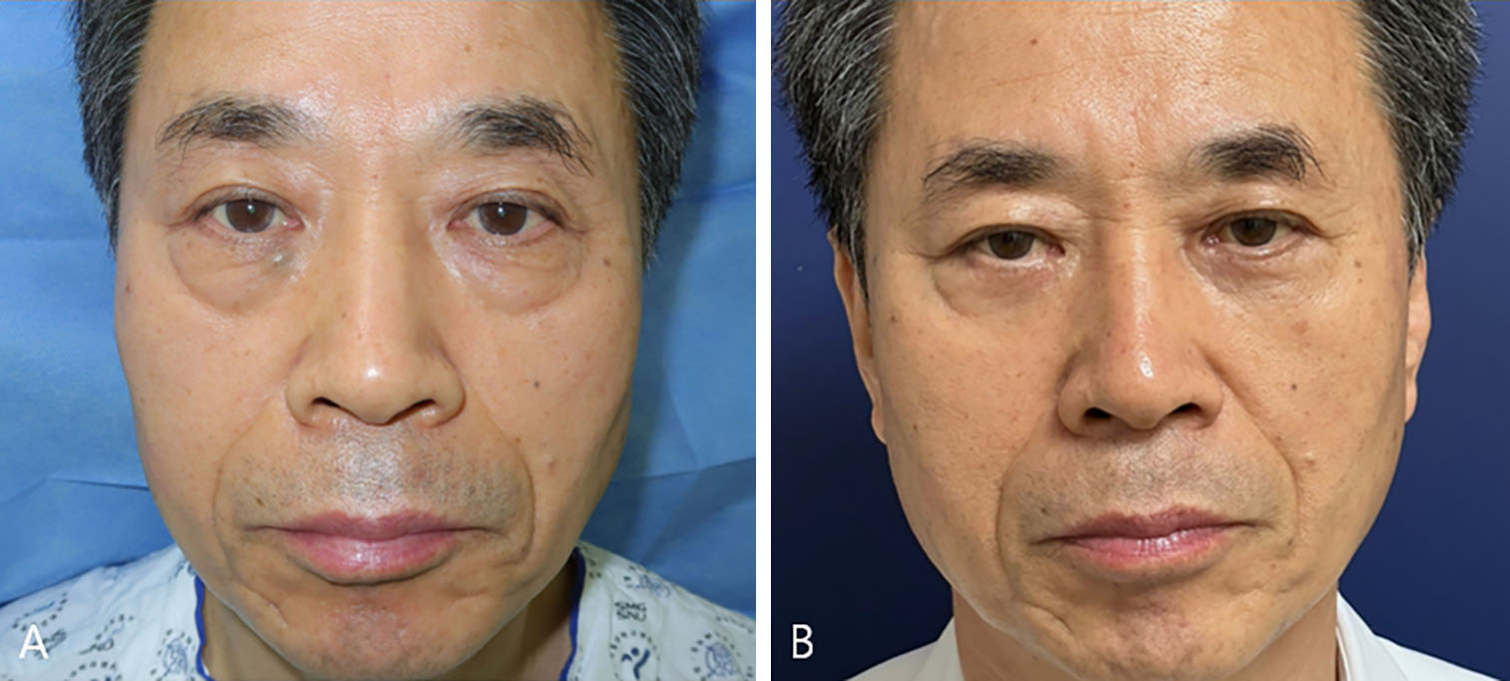
The third case of patient who underwent lower blepharoplasty without mid-face lift. Photographic findings of 74-year-old female patient with baggy lower eyelid and aging process in mid-cheek region. **A** Postoperative photographic finding; 1 year. **B**

A 69-year-old man visited clinic for a baggy lower eyelid and aging process in mid-cheek region. He had no history of facial aesthetic surgical procedure. The patient complained of soft tissue sagging in the anterior cheek and deeper-appearing nasolabial fold. Lower blepharoplasty was performed including resection of redundant fat component without mid-face lift (Fig. 7). At one year after operation, mean changes of MFWS and AMVDS were 1.2 and 1.5. WIZDOM-MC decreased 2.3mm compared to preoperative finding and mean POSAS was 0.9 at 1 year after operation. Patient was very satisfied with the outcome of lower blepharoplasty combined with mid-face lift procedure.

## Discussion

With the increasing emphasis on aesthetic desires driven by societal changes, there has been a growing demand for cosmetic surgeries such as brow lift, blepharoplasty, and mid-face lift. Facial aging, characterized by lower eyelid ptosis and mid-face sagging, can be attributed to various anatomical factors. Several reference papers have explored these underlying causes. According to a previous study, lower eyelid ptosis is primarily a result of age-related changes in the orbital septum, weakening of the orbital retaining ligament, and prolapse of orbital fat [18–20]. Furthermore, several studies highlighted the anatomical changes in the mid-face region, including descent of the malar fat pad, weakening of the retaining ligaments, and resorption of bony support structures, leading to sagging cheeks and nasolabial folds. [21–23]

In this study, lower blepharoplasty was performed sorely utilizing the subciliary incision for baggy lower eyelids resulting from the anatomical changes associated with aging, as previously explained. Subciliary incision lower blepharoplasty offers several advantages compared to the transconjunctival approach. Several previous reports highlight these benefits, providing evidence for the efficacy and favorable outcomes associated with this technique. For instance, Stevens described the subciliary incision technique as providing better access to the lower eyelid fat compartments and allowing for more precise fat removal and repositioning [24]. He emphasized that this approach enables direct visualization of the lower eyelid structures, facilitating precise control over fat removal and achieving a smoother contour. Moreover, May Jr. demonstrated that the subciliary incision technique is particularly effective in addressing lower eyelid skin excess and laxity, allowing for direct excision and skin tightening [25]. Additionally, Haefliger noted that the subciliary incision approach offers the advantage of direct access to the lower eyelid muscle for precise muscle tightening and suspension, which can contribute to improved functional and aesthetic outcomes. [26]

Traditional mid-face lift, also known as a mid-face rejuvenation is a surgical procedure aimed at addressing signs of aging in the mid-face region. It involves repositioning and lifting sagging facial tissues, including the cheeks and lower eyelids, to restore a more youthful and rejuvenated appearance. By subciliary incision, the surgeon access to the underlying facial structures. Once the incisions are made, tissue dissection and repositioning are performed. This may involve tightening and lifting the underlying muscles such as levator labii superioris muscle, as well as manipulating sub-orbicularis oculi fat (SOOF). Although traditional mid-face lift is a comprehensive procedure that can yield effective result; however, it is important to consider some of its drawbacks. One disadvantage is the relatively longer operation time required for traditional mid-face lift compared to less invasive procedures such as thread lifts. The extensive tissue dissection and repositioning involved in the surgery can contribute to increased surgical duration. Additionally, the invasiveness of the procedure may lead to a higher risk of potential complications and side effects. These can include postoperative swelling, bruising, and discomfort. Furthermore, traditional mid-face lift may carry risks associated with general anesthesia, such as adverse reactions or complications related to anesthesia administration.

With the increasing popularity of non-invasive procedures, there has been a growing interest in the use of PDO thread lift for facial rejuvenation [27]. PDO thread lift is performed for facial sagging, wrinkles, and volume loss, leading to overall facial rejuvenation. [8, 28–30] These studies encompass a wide range of applications, including brow lifting, mid-face lifting, jawline contouring, and neck tightening. The non-invasive nature of PDO thread lift procedures offers several advantages. Patients can benefit from minimal downtime and reduced risks compared to traditional surgical methods. Furthermore, the absorbable nature of PDO threads ensures that the results are temporary and allow for adjustments as needed. The threads stimulate collagen production, promoting tissue contraction and enhancing long-term aesthetic outcomes. [29, 30] In this study, we employed PDO thread lift within the subciliary incision for mid-face lift and correction of nasolabial fold wrinkles, as an alternative to the traditional mid-face lift procedure involving extensive dissection.

By performing less invasive procedure using threads, no complications such as hematoma, skin necrosis, or nerve injury were reported. During the procedure, there were concerns about potential postoperative widening of the alar base after surgery. However, we did not observe significant widening of the alar base, and none of our patients reported this issue. To prevent this phenomenon, the author concentrated on the vector of the zygomaticus minor muscle rather than a pulling vector perpendicular to the nasolabial fold. Additionally, to avoid alar deformity, the ends of the PDO threads were carefully placed to neither disturb the plane of the nasalis muscle nor approach the subcutaneous region near the base of the ala. Furthermore, the absence of observed bruising and severe pain immediately following the surgery in patients can be attributed to the utilization of this methods. This can be considered as a reflection of patient satisfaction with the surgical outcomes.

When discussing the advantages of performing ultrasound-guided thread lift procedures based on the anatomical structures of mid-face, several points can be considered from the perspective of stability, efficiency, and prevention of side effects. By utilizing ultrasound guidance during thread lift procedures, real-time visualization of anatomical structures allows for improved the accuracy and precision of thread placement. This can contribute to more predictable and satisfactory outcomes. In addition, the ability to visualize important structures such as blood vessels and nerves helps in avoiding potential damage during the procedure. By minimizing the risk of unintended injuries, ultrasound-guided thread lift procedures can offer increased safety and reduce complications. Furthermore, ultrasound imaging assists in identifying the specific areas of concern, enabling targeted placement of threads to address sagging or wrinkling in a precise manner. As a result, the use of ultrasound-guided techniques can potentially minimize tissue trauma and postoperative swelling, and it may lead to reduced downtime and quicker recovery for patients. However, no significant difference was found in scar assessment between the two surgical methods, which is likely attributed to the anatomical characteristics of the periorbital area where scars are not as noticeable near the lower eyelid. [31]

Mean operation time significantly increased when patients underwent lower blepharoplasty with mid-face lift compared to the lower blepharoplasty-only group. However, despite the additional 15 minutes required for the procedure, there were significant improvements in nasolabial wrinkle reduction, mid-face lift, and overall patient satisfaction observed up to 1 year. The inclusion of this relatively simple 15-minute procedure enabled the achievement of multiple aesthetic advantages.

However, this study has several limitations. Firstly, the sample size included in this retrospective study was small, and the follow-up period of 1 year may not be sufficient to assess long-term surgical outcomes and patient satisfaction. Nevertheless, this study holds significance as it introduces the surgical advantages and stability of combining ultrasound-guided lower blepharoplasty with mid-face lift, which has not been previously explored. The direct lasting effect of mid-face lifting with PDO threads is generally understood to be around two years, though there is ongoing debate about this duration. Nevertheless, this study corroborates that the procedure offers considerable benefits for managing postoperative scars. Furthermore, the longevity of PDO threads might be extended by sustaining the thread lift's effects via fibrosis, as indicated in prior research. [29] Furthermore, there is a need for prospective studies with a larger number of patients. Additionally, all patients in this study underwent the removal of excessive fat, but the surgical outcomes could have varied depending on the amount of fat removed, which was not measured, thus posing a limitation. Moreover, the inclusion of two patients who developed suture granuloma after surgery in the statistical analysis is considered a limitation. Lastly, while this study focused on patient preferences for non-invasive procedures, future research plans to explore the differences in surgical outcomes between traditional mid-face lift and thread based mid-face lift, which were not included in this study.

## Conclusions

The combined approach of lower blepharoplasty and mid-face lift using PDO threads offers a comprehensive solution for facial rejuvenation, resulting in significant improvements in wrinkle reduction, mid-face lift, and patient satisfaction. The use of ultrasound-guided thread lifting, which involves assessing the anatomical structures of the mid-face and performing lifting procedures, is a safe and efficient method. This approach should be considered a promising option for future cosmetic anti-aging surgery.

## Abbreviations

PDO: Polydioxanone
MFWS: Modified Fitzpatrick Wrinkle Scale
AMVDS: Allergan® Midface Volume Deficit Scale
WIZDOM-MC: Width of Inter Zygomatic Distance of the Midface to Medial Canthus
POSAS: Patient and Observer Scar Assessment Scale
SMAS: Superficial Musculo Aponeurotic System
SOOF: Sub-Orbicularis Oculi Fat
